# Astrocytes and glutamate homoeostasis in Alzheimer's disease: a decrease in glutamine synthetase, but not in glutamate transporter-1, in the prefrontal cortex

**DOI:** 10.1042/AN20130017

**Published:** 2013-10-07

**Authors:** Magdalena Kulijewicz-Nawrot, Eva Syková, Alexander Chvátal, Alexei Verkhratsky, José J. Rodríguez

**Affiliations:** *Institute of Experimental Medicine, ASCR, Videnska 1083, 142 20 Prague, Czech Republic; †Department of Neuroscience and Center for Cell Therapy and Tissue Repair, Charles University, Second Medical Faculty, Prague, Czech Republic; ‡Faculty of Life Sciences, The University of Manchester, Manchester M13 9PT, U.K.; §IKERBASQUE, Basque Foundation for Science, 48011 Bilbao, Spain; ∥Department of Neurosciences, University of the Basque Country UPV/EHU, 48940 Leioa, Spain and CIBERNED

**Keywords:** Alzheimer’s disease, astroglia, glial fibrillary acidic protein (GFAP), glutamate transporter-1 (GLT-1), glutamine synthetase, medial prefrontal cortex, Aβ, amyloid β, AD, Alzheimer’s disease, ALS, amyotrophic lateral sclerosis, CNS, central nervous system, EAAT, excitatory amino acid transporter, GABA, γ-aminobutyric acid, GFAP, glial fibrillary acidic protein, GFAP-IR, GFAP immunoreactivity/immunoreactive, GLAST, glutamate aspartate transporter, GLT-1, glutamate transporter-1, GS, glutamine synthetase, GS-IR, GS immunoreactivity/immunoreactive, LTP, long-term potentiation, MDD, major depressive disorder, Nv, numerical density, TS, Trizma base saline

## Abstract

Astrocytes control tissue equilibrium and hence define the homoeostasis and function of the CNS (central nervous system). Being principal homoeostatic cells, astroglia are fundamental for various forms of neuropathology, including AD (Alzheimer's disease). AD is a progressive neurodegenerative disorder characterized by the loss of cognitive functions due to specific lesions in mnesic-associated regions, including the mPFC (medial prefrontal cortex). Here, we analyzed the expression of GS (glutamine synthetase) and GLT-1 (glutamate transporter-1) in astrocytes in the mPFC during the progression of AD in a triple-transgenic mouse model (3xTg-AD). GS is an astrocyte-specific enzyme, responsible for the intracellular conversion of glutamate into glutamine, whereas the removal of glutamate from the extracellular space is accomplished mainly by astroglia-specific GLT-1. We found a significant decrease in the numerical density (Nv, cells/mm^3^) of GS-positive astrocytes from early to middle ages (1–9 months; at the age of 1 month by 17%, 6 months by 27% and 9 months by 27% when compared with control animals) in parallel with a reduced expression of GS (determined by Western blots), which started at the age of 6 months and was sustained up to 12 months of age. We did not, however, find any changes in the expression of GLT-1, which implies an intact glutamate uptake mechanism. Our results indicate that the decrease in GS expression may underlie a gradual decline in the vital astrocyte-dependent glutamate–glutamine conversion pathway, which in turn may compromise glutamate homoeostasis, leading towards failures in synaptic connectivity with deficient cognition and memory.

## INTRODUCTION

Astrocytes in the CNS (central nervous system) act as the primary homoeostatic cells; in addition, astroglia exert overall control over synaptic function by isolating single synapses, thus ensuring the spatial specificity of synaptic inputs and providing synaptic structures with metabolic substrates and glutamine (Hertz and Zielke, 2004; Nedergaard and Verkhratsky, 2012). The latter is indispensable as a precursor for the major excitatory and inhibitory transmitters, glutamate and GABA (γ-aminobutyric acid). Furthermore, astrocytes regulate synaptogenesis and may directly affect synaptic connectivity by releasing neuromediators such as ATP and/or d-serine and by regulating the level of extracellular glutamate (Pfrieger and Barres, 1997; Anderson and Swanson, 2000; Ullian et al., 2001; Eroglu et al., 2008; Stipursky et al., 2011). Astrocytes are also capable of secreting antioxidants and trophic factors, for example BDNF (brain-derived neurotrophic factor), glial-derived neurotrophic factor and neurotrophins (Zaheer et al., 1995; Vargas et al., 2006).

Glutamate is the principal excitatory neurotransmitter in the mammalian CNS and it is fundamentally important for cognition, memory and learning (Fonnum, 1984; Collingridge and Lester, 1989; Headley and Grillner, 1990). Glutamatergic signalling is responsible for synapse formation and elimination, as well as for the regulation of neural cell migration, differentiation, survival and death (Komuro and Rakic, 1993; Durand et al., 1996; Danbolt, 2001). Long-lasting exposure to high levels of glutamate cause excitotoxicity (Greenamyre et al., 1988; Walton and Dodd, 2007), which is involved in various brain pathologies such as ALS (amyotrophic lateral sclerosis), Huntington's disease, AD (Alzheimer's disease), epilepsy, ischemia and trauma (Won et al., 2002; Hynd et al., 2004; Walton and Dodd, 2007; Nedergaard et al., 2010; Verkhratsky et al., 2012).

Astrocytes are principal protectors against excitotoxicity and are key elements in glutamate homoeostasis and metabolism (Martinez-Hernandez et al., 1977; Erecinska and Silver, 1990; Westergaard et al., 1995). Glutamate clearance is maintained mainly by astroglia-specific glutamate transporters, the EAAT1/2 (excitatory amino acid transporters 1 and 2), classified, in rodents, as GLAST (glutamate aspartate transporter) and GLT-1 (glutamate transporter-1) (Rothstein et al., 1994; Danbolt, 2001). EAAT2/GLT-1 is the most abundant form of glutamate transporter in the CNS, responsible for approximately 90% of glutamate uptake in the cortex and hippocampus (Lehre et al., 1995; Berger and Hediger, 1998; Danbolt, 2001; Yang and Rothstein, 2009). Glutamate taken up by astrocytes is converted into glutamine by GS (glutamine synthetase) and then transported back to neurons, where it is used for conversion into glutamate and GABA [glutamate–glutamine shuttle and GABA–glutamine shuttle (Walton and Dodd, 2007)].

AD is a neurodegenerative disorder characterized by defined histopathological hallmarks [intracellular and extracellular aggregation of Aβ (β-amyloid) and intraneuronal accumulation of hyperphoshorylated Tau protein] and manifested by progressive memory loss and cognitive decline (Selkoe, 2001; Cummings, 2004; Battaglia et al., 2007). Altered glutamatergic signaling, resulting from alterations in the expression of glutamate transporters (vesicular glutamate transporters VGLUT1/2 EAAT1/GLAST and EAAT2/GLT-1) and relevant enzymes [GS, GSLP (GS-like protein), PAG (phosphate-activated glutaminase), GDH (glutamate dehydrogenase)], as well as changes in the protein levels of specific subunits of ionotropic and metabotropic glutamate receptors, is likely to be involved in the neuronal pathology observed in AD (Miguel-Hidalgo et al., 2002; Burbaeva et al., 2005; Revett et al., 2012). In patients diagnosed with AD, deficits in NMDA (*N*-methyl-d-aspartate)-dependent forms of neocortical LTP (long-term potentiation), revealed by paired associative stimulation, have been observed, indicating altered glutamatergic synaptic plasticity (Stefan et al., 2000; Battaglia et al., 2007).

Despite the broad research on glutamate metabolism failure in AD, there is still lack of strong data and common agreement about the role of molecules involved. Here, we performed an in-depth analysis of the main components of astroglia-dependent glutamate homoeostasis, GS and GLT-1, in a triple transgenic mouse model of AD (3xTg-AD). This animal model develops Aβ plaques and neurofibrillary tangles in a sequential manner (Oddo et al., 2003b). Apart from histopathological hallmarks, 3xTg-AD mice show functional and cognitive impairments including LTP, spatial memory and long-term memory deficits, all being manifested in an age-related manner (Oddo et al., 2003a, 2003b). 3xTg-AD mice also show a certain level of neuronal loss accompanied by spine loss on dystrophic dendrites making the model relevant for studying alterations in synaptic transmission (Bittner et al., 2010; Fuhrmann et al., 2010).

Considering our results and general broad knowledge of astrocytic role in glutamate–glutamine conversion mechanism, we may hypothesize that changes in GS expression may influence glutamate homoeostasis and glutamine supply to neurons, leading towards failures in synaptic connectivity and transmission, affecting brain functions such as mood, cognition, and memory, which all are impaired in AD.

## MATERIALS AND METHODS

This study was performed in accordance with the European Union Directive of 22nd September 2010 (2010/63/EU) regarding the use of animals in research and was approved by the Ethical Committee of the Institute of Experimental Medicine of the Academy of Sciences of the Czech Republic, Prague, Czech Republic. All efforts were made to reduce the number of animals.

### Animals

Experiments were performed on male 3xTg-AD mice and their background-matching controls as described in detail previously and above in the introduction (Oddo et al., 2003a, 2003b; Rodriguez et al., 2008; Rodriguez et al., 2009a).

### Immunohistochemical analysis

#### Fixation and tissue processing

3xTg-AD animals of different age groups and their equivalent non-transgenic (Non-Tg) controls (1, 6, 9 and 12 months; *n*=4–7) were intraperitoneally anesthetized with sodium pentobarbital (50 mg/kg). All subsequent procedures were done in the same way as described previously (Kulijewicz-Nawrot et al., 2012). Briefly, brains were then removed and cut into 4 mm coronal slabs of tissue containing the mPFC, post-fixed in 2% paraformaldehyde for 24 h and cut into 40–50 μm thick coronal sections using a vibrating microtome (MICROM HM 650 V, Thermo Scientific). For immunohistochemistry, coronal sections at levels 1.98/1.54 mm anterior to Bregma were selected, according to the mouse brain atlas of Paxinos and Franklin (Paxinos and Franklin, 2004).

#### Antibodies

A mouse antiserum generated against GS (anti-GS; Millipore, catalogue number MAB302) was used for the determination of GS-positive astrocytes. An IgG fraction of rabbit anti-GFAP (glial fibrillary acidic protein; Sigma–Aldrich, catalogue number G9269) was used for the determination of the glial cytoskeleton and comparison with GS labelling distribution. The immunolabelling pattern we obtained with this antibody was equivalent to that obtained previously in different brain regions (Wilhelmsson et al., 2004; Olabarria et al., 2011). The specificity of the antibody has been reported previously using immunohistochemistry and Western blotting (Pike et al., 1996; Wilhelmsson et al., 2004; Sen et al., 2011). To assess non-specific background labeling or cross-reactivity between antibodies derived from different host species, a series of control experiments were performed. The omission of either the primary or secondary antibody resulted in a total absence of target labeling (data not shown).

#### Immunohistochemistry

The procedure for immunohistochemistry was the same as described previously [for details see (Olabarria et al., 2011; Yeh et al., 2011; Kulijewicz-Nawrot et al., 2012)]. After initial tissue preparation, for single labeling the sections were incubated for 48 h at room temperature with the primary antibody (mouse anti-GS, 1:500 dilution, catalogue number MAB302, Millipore), then after rinsing in 0.1 M TS (Trizma base saline) for 30 min the sections were incubated in a 1:200 dilution of biotinylated horse anti-mouse IgG (Vector Laboratories) for 1 h at room temperature (20–25°C). Sections were rinsed in 0.1 M TS for 30 min, followed by incubation for 30 min in avidin–biotin peroxidase complex (Vector Laboratories). The peroxidase reaction product was visualized by incubation in a solution containing 0.022% DAB (3,3′diaminobenzidine, Sigma–Aldrich) and 0.003% H_2_O_2_ for 2.5 min as described previously (Rodriguez et al., 2008; Rodriguez et al., 2009a; Olabarria et al., 2011). The final steps, included dehydrating in ascending concentrations of ethanol followed by xylene, before being permanently coverslipped with entellan (Merck).

For dual immunofluorescence labelling, the sections were incubated for 48 h at room temperature in a primary antibody cocktail containing: (i) mouse anti-GS (1:1000 dilution) and (ii) rabbit anti-GFAP (1:5000 dilution) simultaneously. GS and GFAP were detected in a consecutive manner by incubation with Alexa Fluor® 594-conjugated goat anti-mouse IgG (Invitrogen) and FITC-conjugated goat anti-rabbit IgG (Jackson Immunoresearch) respectively. After immunofluorescence labeling, the sections were rinsed with 0.1 M TS for 30 min and permanently mounted in an aqueous medium (Vectashield, Vector Laboratories).

#### GS-immunoreactive cell density

We determined the numerical density (Nv; number of cells/mm^3^) of GS-positive astrocytes at 1, 6, 9 and 12 months of age in both 3xTg-AD and Non-Tg mice in the mPFC. For this, three to four representative non-consecutive coronal sections throughout the mPFC at levels 1.98/1.54 mm from Bregma were quantified, accounting for an approximate volume of 24000000 μm^3^. GS-positive astrocytes were intensely labeled against a lighter background, which made them easy to identify with an equal chance of being counted. A single observer determined the number of GS-positive astrocytes in a blinded fashion to keep the counting bias to a minimum.

#### Measurements of GS-immunoreactive cell domain and cell body surface area

We used ImageJ software to measure the representative cell domain and cell body surface area in each age group (number of cells=15 in both controls and 3xTG-AD animals). For this purpose higher magnification micrographs (objective 40×) obtained with light microscopy (Zeiss Observer.D1) were analyzed.

#### Co-localization of GS-IR (GS immunoreactivity/immunoreactive) and GFAP-IR (GFAP immunoreactivity/immunoreactive) astrocytes

To determine the co-localization of GS-IR and GFAP-IR astrocytes, representative higher magnification stacks of images throughout the mPFC region of control and 3xTg-AD animals (*n*=4 in both cases) were taken using a confocal microscope (Zeiss LSM 5 DUO) at 0.2 μm z-step. Both GS-IR and GFAP-IR cells were imaged at the same time and then counted in an approximate area of 40500 μm^2^ in sections of 40 μm thickness.

### Western blot analysis

#### Tissue processing

Transgenic and control animals of different age groups 1, 6, 9 and 12 months; *n*=5–7) were euthanized by cervical dislocation. Brain tissue samples containing the mPFC were collected immediately and lysed with 100 μl of STEN lysis buffer [50 mM Tris (pH 7.6), 150 mM NaCl, 2 mM EDTA and 1% Triton X-100 (Sigma–Aldrich)] with protease inhibitors (Complete mini, Protease Inhibitor Cocktail Tablets, Roche) on ice for 30 min. When ready to use, the lysates were centrifuged (21000 ***g*** for 5 min) and the supernatant was transferred to a new eppendorf tube.

#### Western blotting

The protein concentrations of the brain tissue lysates were determined using the Bradford method (Bio-Rad) (Bradford, 1976). Samples containing 20 μg of protein and 1× Laemmli buffer (Laemmli, 1970) were boiled at 95°C for 4–5 min. Samples were loaded together with 5 μl of protein marker (pre-stained Protein Ladder, Page Ruler, Fermentas) and run on SDS/PAGE {12% gels [30% acrylamide:bisacrylamide (37,5:1), 1.5 M Tris (pH 8.8), 10% APS (ammonium persulfate), 10% SDS and 0.1% TEMED (tetramethylethylene-diamine)]}. The gels were submerged into 1× running buffer (25 mM Tris base, 119 mM glycine and 1% SDS) and run initially at 100 V until the samples passed the stacking gel, then at 150 V until the mercaptoethanol dye reached the bottom of the gel. After electrophoresis, the proteins were transferred on to a nitrocellulose membrane in an electrical field in order to immobilize them in a specially designed chamber (Bio-Rad). Prior to transfer, the gel and nitrocellulose membrane were dunked in 1× transfer buffer [25 mM Tris base, 119 mM glycine and 20% methanol (pH 7.6)] and run at 400 mA (constant) for 120 min.

After transfer, to prevent the non-specific binding of the primary and secondary antibodies, membrane blocking was performed in a blocking solution consisting of 5% non-fat dried skimmed milk dissolved in TBST buffer [Tris-buffered saline- Tween 20 (10 mM Tris base, 100 mM NaCl and 0.1% Tween 20, pH 7.6)]. Blots were incubated for 1 h at room temperature with agitation.

#### Antibodies

Blots were probed with the following antibodies: mouse anti-GS (1:20000 dilution) (Millipore, catalogue number MAB302), rabbit anti-GLT-1 [GLT-1/EAAT2 polyclonal antibody (1:1000 dilution)] (Cell Signaling Technology, catalogue number 3838) and mouse anti-β-actin monoclonal antibody (1:20000 dilution) (Sigma–Aldrich, catalogue number A2228). Staining for β-actin was performed as a control of equal protein loading. The specificity of the antibodies has been reported previously using Western blotting (Gimona et al., 1994; Tanaka et al., 1997; Amara and Fontana, 2002; Christie et al., 2007; Sen et al., 2011).

#### Protein detection and band analysis

The primary antibodies were diluted in the same blocking buffer (5% non-fat dried skimmed milk/TBST), and the membranes were incubated for 1 h (in the case of anti-GS and anti-β-actin antibodies) or 2 h (in the case of the anti-GLT-1 antibody) at room temperature. Following the incubations, the membranes were washed three times in TBST at room temperature with agitation for 15 min to remove residual primary antibodies. Blots were then probed with HRP (horseradish peroxidase)-conjugated secondary antibodies (goat anti-mouse IgG, 1:15000 dilution; goat anti-rabbit IgG 1:20000 dilution; Jackson Immunoresearch) and incubated for 1 h with agitation at room temperature. Finally, the membranes were washed three times as described above.

Visualization of the secondary antibodies was achieved with ECL (enhanced chemiluminescence) substrate and incubated for 5 min in the dark at room temperature and subsequently exposed to XBM X-ray film (Retina, Fotochemische Werke). After scanning the images, ImageJ free software was used to quantify the intensity of the bands. The ratio of GS or GLT-1 to β-actin, used as a loading control, was first assessed. In order to perform the comparison across different Western blots, an internal control was always included on each blot as a reference point regarding the GS or GLT-1/β-actin ratio.

### Statistical analysis

An unpaired *t* test was used to examine differences in the GS-positive cell number and surface area as well as in the relative ratios of GS or GLT-1 to β-actin between the 3xTg-AD and Non-Tg animals. A linear regression test was used to analyze the relationship between the age and relative ratios of GS to β-actin in the 3xTg-AD and Non-Tg animals. Data are expressed as means±S.E.M.. The data were analyzed using GraphPad Prism (GraphPad Software).

## RESULTS

**Figure 1:**
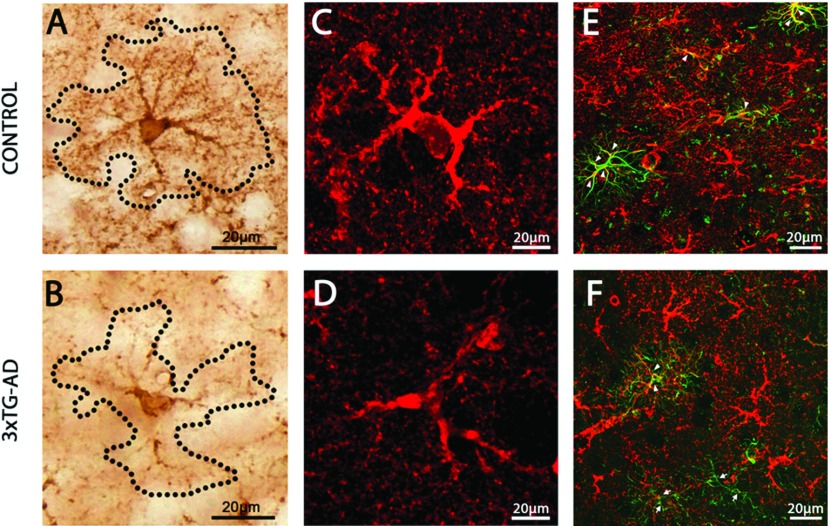
The astrocytic phenotype of GS-IR astrocytes and their co-localization with GFAP are altered in AD mice Light and confocal images of GS-IR astrocytes in the mPFC of Non-Tg control animals (**A** and **C**) and 3xTg-AD mice (**B** and **D**), illustrating the difference in astrocytic morphology and appearance. (**E** and **F**) Confocal images demonstrating astrocytic GFAP and GS co-expression, which is much reduced in 3xTg-AD animals. The majority of GFAP-IR astrocytes from control mice do co-express GS (**E**; arrowheads). In contrast, even if some 3xTg-AD GFAP-IR astroglia show co-expression with GS (**F**; arrowheads), many GFAP-IR astrocytes fail to express both proteins at the same time (**F**; arrows).

Cortical GS-IR astrocytes were uniformly distributed in both the superficial and deep layers of the mPFC. GS-IR astrocytes showed clear labeling of their primary and distal processes, thus faithfully delineating the astrocytic domains (Figures 1A and 1C). GS-IR astrocytes showed typical characteristics of protoplasmic astrocytes with multiple elaborated processes emanating from the cell somata (Figure 1A). In control animals the astrocytic cell bodies were well defined and almost perfectly round in shape. In 3xTg-AD animals, the GS-IR cell bodies were also clearly outlined, but slightly less spherical and markedly smaller (see Figures 1B and 1D, and below). In 3xTg-AD animals, GS-positive astrocytes also had less branching in both their main and secondary processes (Figures 1B and 1D). The processes were directed in a random fashion, with thin extensions oriented in various directions.

As revealed by co-staining, there was a different tendency to co-express GFAP and GS in mPFC astrocytes in control and 3xTg-AD mice. In general, in controls and transgenic animals three subpopulations of astrocytes were identified: GS-IR, GFAP-IR and a population expressing both proteins (GS/GFAP-IR) (Table 1). The majority of astroglial cells in the region of interest in control animals were GS-IR (72.90%), while the population of GFAP-IR astrocytes constituted 9.03%. In 3xTg-AD mice 82.50% of astrocytes were GS-IR, whereas 12.5% were GFAP-IR. The most interesting difference was observed in the case of astrocytes co-expressing GS and GFAP (GS/GFAP-IR). In 3xTg-AD animals just 5% expressed both proteins simultaneously (Figure 1F), whereas up to 18.07% of astrocytes in control animals did so (Figure 1E).

**Table 1:** Different distribution of astrocytic subpopulations in the mPFC Numbers represent changes in numerical density (cells/mm^3^) in Non-Tg and 3xTg-AD animals (±S.E.M.).

Population	Non-Tg	3xTg-AD
GS-IR	17423±388	15265±918
GFAP-IR	2158±308	2312±388
GS/GFAP-IR	4317±752	925±178
Total mean Nv	23899±1108	18503±1153

### Reduced Nv of GS-IR astrocytes in the early and middle stages of AD

**Figure 2:**
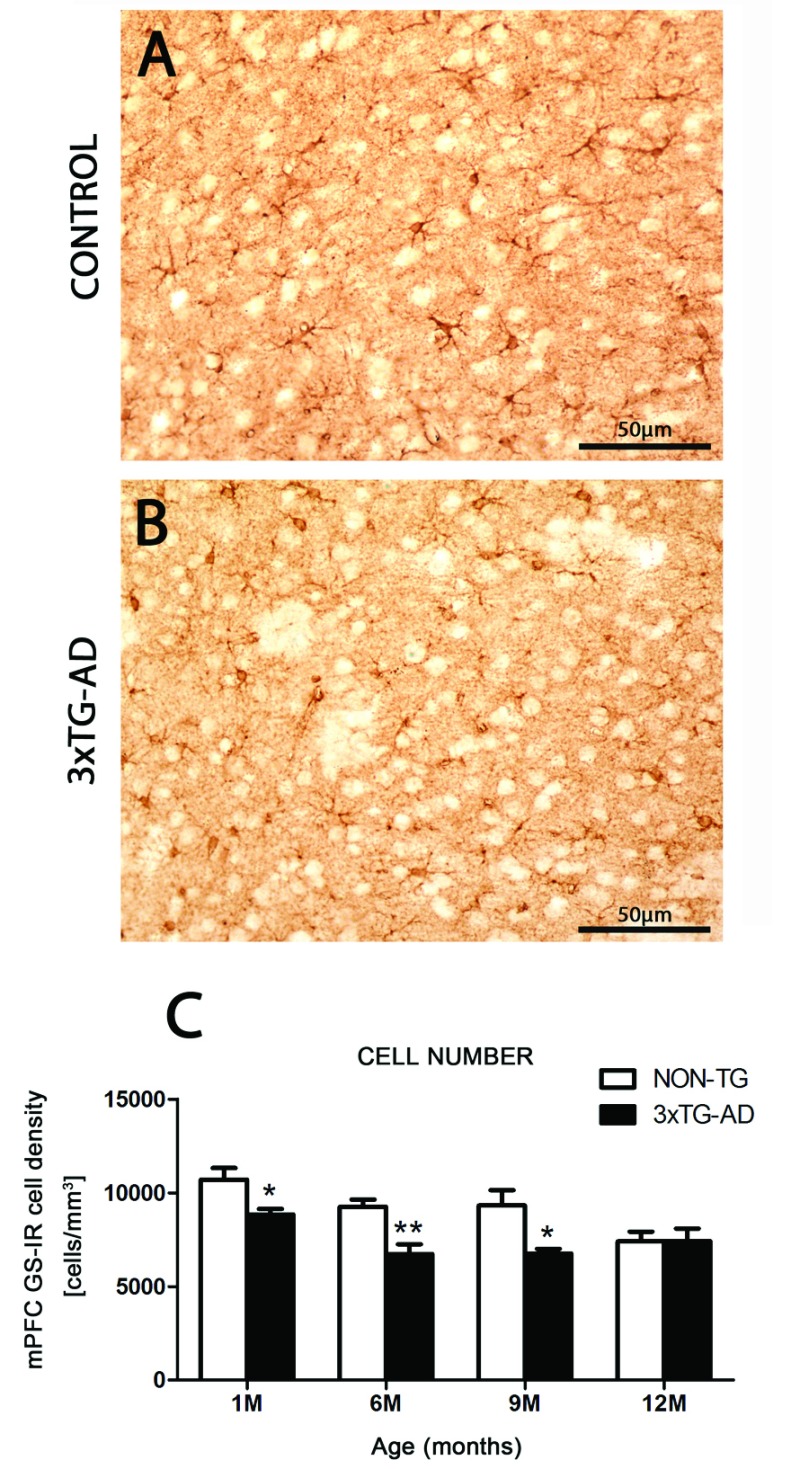
Differential distribution and number of GS-IR astrocytes between control and transgenic mice Light micrographs showing the distribution of GS-IR astrocytes within the mPFC in Non-Tg control (**A**) and 3xTg-AD (**B**) animals. (**C**) Histograms showing the numerical density (number of cells/mm^3^) of GS-IR cells in the mPFC of 3xTg-AD and Non-Tg controls. Bars represent means±S.E.M.

From 1 month of age, a significant reduction appeared in the Nv of GS-IR cells in 3xTg-AD mice in the mPFC, when compared with control animals (17.38%; *P*=0.0337). This reduction was sustained and further progressed through more advanced ages, being 27.21% at 6 months (*P*=0.0079) and 27.52% at 9 months (*P*=0.0252) (Figure 2 and Table 2).

**Table 2:** Reduced Nv of GS-IR astrocytes in the mPFC during early and middle stages of AD Numbers represent changes in numerical density (cells/mm^3^) in the Non-Tg and 3xTg-AD animals at different ages (±S.E.M.).

Age (months)	Non-Tg	3xTG-AD
1	10706±609	8845±299
6	9250±384	6732±517
9	9317±827	6753±254

The decrease in GS-IR cell numbers in 3xTg-AD animals was paralleled by a decrease in the surface area of the cell bodies, which reached significant levels at the age of 1 and 6 months (by 40.69%, *P*<0.0001, and by 39.24%, *P*<0.0001 respectively). The decrease in the size of the GS-IR astroglial profiles was associated with a shrinkage of the astrocytic domain, parallelled with less branchy processes as compared with controls (at 1 month by 46.71%, *P*<0.0001, and at 6 months by 41.28%, *P*<0.0001).

### GS expression decrease from the early to middle stages of AD

**Figure 3:**
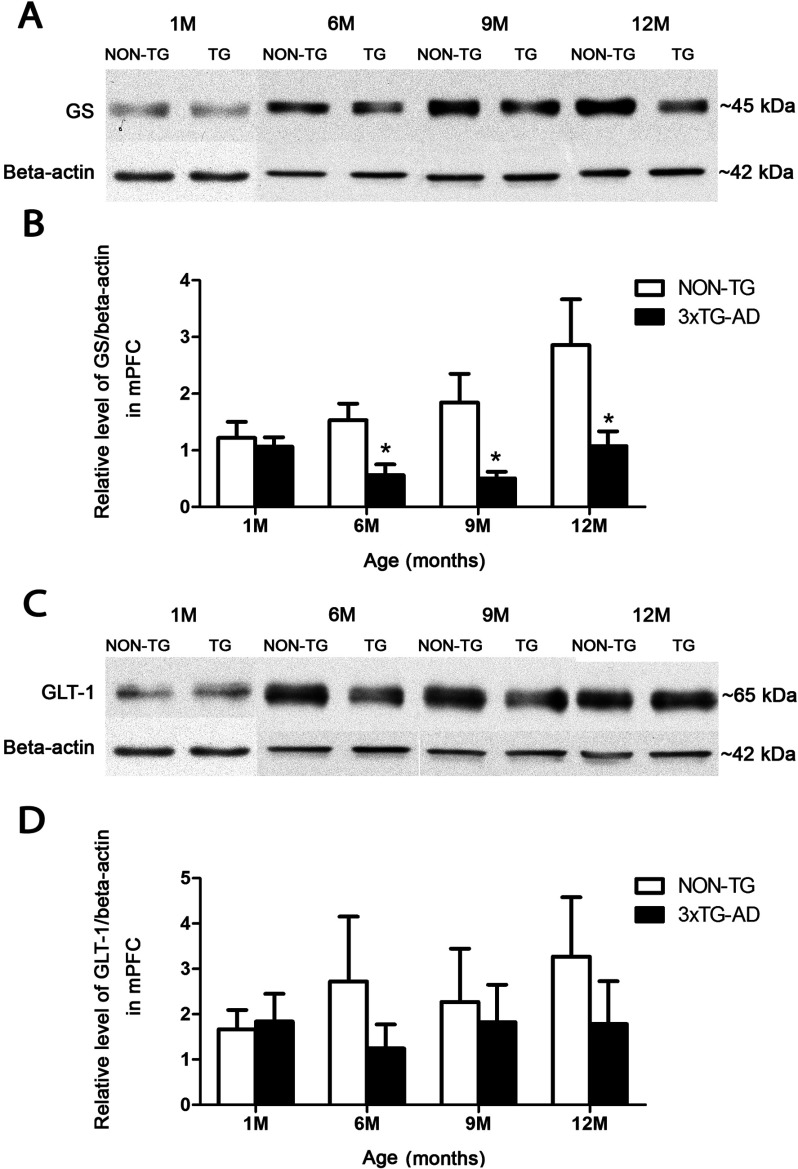
Decrease in astrocytic GS expression, but not in GLT-1 Histograms and representative Western blots showing the relative levels of GS (**A** and **B**) and GLT-1 (**C** and **D**) in the mPFC of 3xTg-AD mice and Non-Tg controls. Bars represent means±S.E.M.

**Figure 4:**
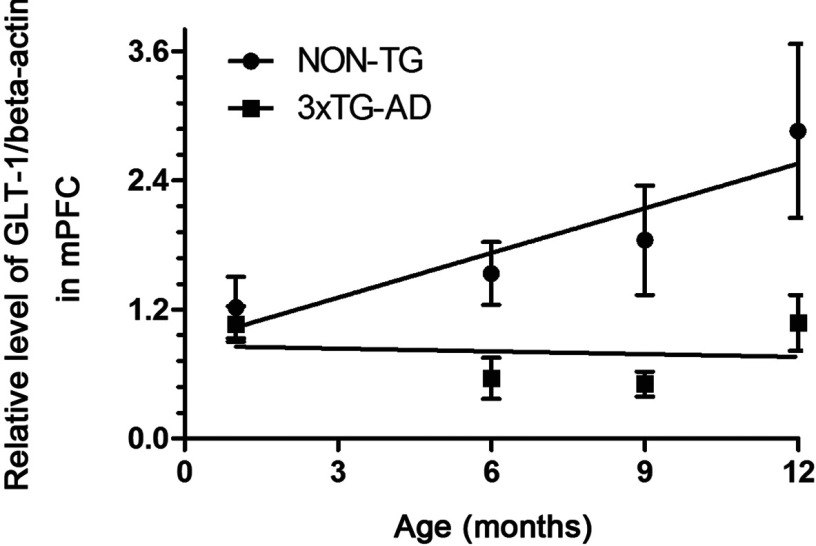
Lack of positive correlation between the age and relative levels of GS Linear regression analysis showing no statistically significant relation between the age and relative levels of GS/β-actin in both Non-Tg and 3xTg-AD animals. Bars represent means±S.E.M.

As determined by Western blots, GS expression was significantly decreased in 3xTg-AD animals compared with controls at both middle and advanced stages of AD (Figure 3A). A significant decrease was observed at 6 months of age (0.561±0.191 compared with 1.534±0.291; *P*=0.021), at 9 months of age (0.507±0.116 compared with1.843±0.509; *P*=0.0444) and at 12 months of age (1.076±0.260 compared with 2.857±0.807; *P*=0.0364) (Figure 3B). After performing linear regression analysis, no statistically significant positive correlation between the age value and relative levels of GS/β-actin was found neither in Non-Tg nor in 3xTG-AD animals (*r*^2^=0.8347, *P*=0.0864, and *r*^2^=0.0166, *P*=0.8713 respectively) (Figure 4).

### GLT-1 remains stable during the progression of AD

Astrocytic GLT-1 expression in 3xTg-AD mice showed no significant difference at any ages when compared with control animals (Figure 3C). Nevertheless, at any given point there was a slight reduction in GLT-1 levels, concomitant with the significantly reduced GS expression (Figure 3D).

## DISCUSSION

In the present study we found a significant decrease in the number of GS-IR astrocytes as well as in the expression of GS protein in astrocytes from the mPFC in the 3xTg-AD animal model. This decrease occurred at early ages, well before pathological and behavioural alterations appeared. A decrease in GS expression was also present in 12-month-old animals, when robust intracellular Aβ accumulations, with a tendency to form extracellular deposits, are clearly visible (Kulijewicz-Nawrot et al., 2012). The decrease at that age in 3xTg-AD animals was revealed by Western blot analysis, but not by the GS-IR cell number (unchanged at 12 months). The lack of a significant difference is due to the simultaneous decrease in the number of GS-IR astrocytes in control animals at 12 months. No significant changes in the expression of GLT-1 were found, although it showed some tendency to decline. Our results indicate disturbances in the glutamate–glutamine cycle, which start early during disease progression in the mPFC, in contrast with the hippocampus, where a decrease in GS-IR astrocytes was observed at 12–18 months of age (Olabarria et al., 2011). When analyzing the astrocytes in the mPFC, we found three different subpopulations of astroglia: GS-IR, GFAP-IR and GS/GFAP-IR. In line with our results, Robinson in his study on the human cortex described complementary populations of astrocytes, GFAP-IR and GS-IR, with quite a heterogeneous regional distribution (Robinson, 2000). The novelty in our case is the presence of the third GS/GFAP-IR population of astrocytes, which further supports the idea of a strong heterogeneity among astrocytes, both morphological and functional (Kimelberg, 2004; Matyash and Kettenmann, 2010; Verkhratsky, 2010; Theis and Giaume, 2012). As an example, this heterogeneity is manifested by the different functional behaviour of astrocytes in the case of injury, by an increase in the co-expression of GFAP and GS and/or by the expression of one of them *de novo* (Humphrey et al., 1997; Walz and Lang, 1998).

Previously, we hypothesized that astrocytic atrophy is involved in AD pathology and may be responsible for a decrease in metabolic support for neurons as well as an altered synaptic environment, including neurotransmitter inactivation and homoeostasis (Rodriguez et al., 2009b; Heneka et al., 2010; Verkhratsky et al., 2010; Rodriguez and Verkhratsky, 2011).

Glutamate is the principal excitatory neurotransmitter in the CNS, and its metabolism depends on astrocytes (Fonnum, 1984; Anderson and Swanson, 2000; Walton and Dodd, 2007). In the human cortex GS is present in astrocytes in all layers, with higher immunoreactivity in layers 1–4; the end-feet covering blood vessels are much less reactive for GS (Robinson, 2000, 2001). Changes in glutamine synthetase have been found in many brain disorders. Up-regulated GS expression was reported in vascular dementia and ALS (Tumani et al., 1999), whereas reduced GS activity was found in hepatic encephalopathy, spinocerebellar atrophy, schizophrenia and epilepsy (Lavoie et al., 1987; Smith et al., 1991; Kish et al., 1994; Le Prince et al., 1995; Burbaeva et al., 2003; van der Hel et al., 2005; Steffek et al., 2008).

Considering the relation of Aβ to GS, the observed reduction in astrocytic enzyme expression in AD patients is not topographically associated with aggregated Aβ, suggesting no obvious correlation between Aβ presence and changes in the expression of GS (Robinson, 2001).

The decreased expression of GS was found at 18 months in the hippocampus of 3xTg-AD animals, when the Aβ plaques are abundant (Olabarria et al., 2011). Here, we demonstrate significant alterations in mPFC GS-IR astrocytes already at the age of 1 month, when no Aβ is detected, and decreased GS protein expression manifested from 6 months onwards in 3xTg-AD mice, which could suggest ongoing metabolic pathology. In line with our results, several research groups have reported decreased concentrations of GS in patients with AD, showing a similar reduction in GS and glutamine levels in the initial stages of AD, suggesting that dysfunction of the glutamate–glutamine cycle is an early event in the progression of the disease (Smith et al., 1985; Csernansky et al., 1996; Jimenez-Jimenez et al., 1998; Robinson, 2001). Also, magnetic resonance spectroscopy at 0.5 T in an *in vivo* study confirmed reduction in levels of glutamate and glutamine in AD patients (Antuono et al., 2001).

Altered glutamatergic transmission in the mPFC can be linked to depression (Miguel-Hidalgo et al., 2010). Based on current research data, a strong association exists between depression and dementia, including AD (Caraci et al., 2010). As revealed by microarray and Western blot analyses, the expression of GS is significantly lower in depressed patients, further corroborating the fundamental importance of astrocytes to the disorder (Choudary et al., 2005; Miguel-Hidalgo et al., 2010).

According to our previous study, a crucial feature of the mPFC is the atrophy of GFAP-positive astrocytic profiles, observed before the typical histopathological onset of the disease (Kulijewicz-Nawrot et al., 2012). This atrophy is further corroborated by reduced GS-IR profiles. Similarly, in studies of the prefrontal cortex of patients with schizophrenia and MDD (major depressive disorder), reductions in GS and GFAP expression have been reported (Steffek et al., 2008; Miguel-Hidalgo et al., 2010).

EAAT2/GLT-1 is the most common glutamate transporter throughout the CNS. It is expressed mainly in the forebrain, striatum, hippocampus and spinal cord, in contrast with GLAST, whose main site of action is in the cerebellum, the retina or the circumventricular organs (Yang and Rothstein, 2009). The down-regulation of EAAT1 and EAAT2 was found in patients suffering from MDD (Miguel-Hidalgo et al., 2010). Beckstrom and colleagues in their elegant study of AD patients with ages from 69 to 94 (Beckstrom et al., 1999) claim individual differences in the levels of glutamate transporters, thus rejecting a straightforward correlation between reduced glutamate transporter expression and AD (Li et al., 1997). In the present study we found GLT-1 expression in the mPFC to be generally unchanged, which can suggest the preservation of glutamate uptake or possible differences in transporter expression between subjects. Our results are in line with Beckstrom's findings highlighting variability in transporter expression and suggesting a common insight into cognitive function decline during severe brain diseases associated with astrocyte alterations and malfunction.

The fact that cognitive deficits are detected long before the clinical diagnosis of AD is made is becoming commonly accepted (Coleman et al., 2004). Synaptic loss and impaired synaptic connectivity have been demonstrated to be causal for cognitive decline also in frontotemporal dementia and normal aging (Lipton et al., 2001; Uylings and de Brabander, 2002). The decrease in synaptic strength and/or connectivity may be a result of altered neurotransmitter cycling, from synthesis to reuptake, a conversion mechanism and vesicle trafficking (Yao and Coleman, 1998; Yao et al., 2003). We believe that structural changes in astrocytes affect synaptic performance (Verkhratsky et al., 2010; Nedergaard and Verkhratsky, 2012). The observed GS deficiency can account for the shortage of glutamine for neurons due to a distorted glutamate–glutamine cycle, resulting in altered synaptic transmission (Antuono et al., 2001). As was shown in similar study, loss of GS was detected in the perisynaptic regions of the neuropil and in astrocytic endfeet, where glutamate transporters are also located (Schmitt et al., 1997; Robinson, 2001). Considering our previous results showing a decrease in the GFAP-IR in astroglia in AD (Olabarria et al., 2010; Rodriguez and Verkhratsky, 2011; Kulijewicz-Nawrot et al., 2012) we may suggest that it is indeed the down-regulation of GFAP which may trigger/force the changes in GS distribution and expression level. Those changes can account for the early as well as the late cognitive impairments in the mPFC region, including personality changes and memory formation (Heidbreder and Groenewegen, 2003). Knowing that GS is highly sensitive to oxidation, one possibility could be usefulness of antioxidant therapy, although there is still lack of consistent data on the topic (Schor, 1988; Vina et al., 2011; Teixeira et al., 2013).

To conclude, our results highlight the crucial role of astrocytes in maintaining metabolic stability within the synaptic and neuronal environments, which makes them a therapeutic target in the prevention as well as the treatment of AD, by maintaining and activating their key homoeostatic role.
